# Application of artificial intelligence in nuclear medicine and molecular imaging: a review of current status and future perspectives for clinical translation

**DOI:** 10.1007/s00259-022-05891-w

**Published:** 2022-07-09

**Authors:** Dimitris Visvikis, Philippe Lambin, Kim Beuschau Mauridsen, Roland Hustinx, Michael Lassmann, Christoph Rischpler, Kuangyu Shi, Jan Pruim

**Affiliations:** 1 LaTIM, INSERM, UMR 1101, University of Brest, Brest, France; 2 The D-Lab, Department of Precision Medicine, GROW – School for Oncology, Maastricht University Medical Center (MUMC +), Maastricht, The Netherlands; 3 Department of Radiology and Nuclear Medicine, GROW – School for Oncology, Maastricht University Medical Center (MUMC +), Maastricht, The Netherlands; 4 Center of Functionally Integrative Neuroscience and MindLab, Department of Clinical Medicine, Aarhus University, Aarhus, Denmark; 5 Department of Nuclear Medicine, University of Bern, Bern, Switzerland; 6 GIGA-CRC in Vivo Imaging, University of Liège, GIGA, Avenue de l’Hôpital 11, 4000 Liege, Belgium; 7 Klinik Und Poliklinik Für Nuklearmedizin, Universitätsklinikum Würzburg, Würzburg, Germany; 8 Department of Nuclear Medicine, University Hospital Essen, University of Duisburg-Essen, Essen, Germany; 9 Department of Informatics, Technical University of Munich, Munich, Germany; 10 Medical Imaging Center, Dept. of Nuclear Medicine and Molecular Imaging, University Medical Center Groningen, University of Groningen, Groningen, The Netherlands

**Keywords:** Artificial intelligence, Nuclear medicine, Molecular imaging

## Abstract

Artificial intelligence (AI) will change the face of nuclear medicine and molecular imaging as it will in everyday life. In this review, we focus on the potential applications of AI in the field, both from a physical (radiomics, underlying statistics, image reconstruction and data analysis) and a clinical (neurology, cardiology, oncology) perspective. Challenges for transferability from research to clinical practice are being discussed as is the concept of explainable AI. Finally, we focus on the fields where challenges should be set out to introduce AI in the field of nuclear medicine and molecular imaging in a reliable manner.

## Introduction

The applications of artificial intelligence (AI) in healthcare are potentially numerous, clearly going beyond the field of medical imaging alone. AI is not a new scientific discipline, having its origins in the Dartmouth conference of 1956 [1]. Although multiple layer perceptrons (MLPs), considered as the origin of “deep” convolutional neural networks (CNNs), were studied since the 70’s, major developments came in the 90’s [2]and early 00’s [3] concerning the learning rules establishing how weights in MLP can be updated. AlexNet [4], winning in 2012 the ImageNet competition performing visual object recognition from photographs, introduced a major breakthrough in neural network performance bringing AI to the forefront of interest on computer vision and imaging applications. A more detailed historical overview can be found elsewhere [5]. Growing numbers of patients, higher demands for quality like early detection and personalized therapies and an increasing workload for medical and nursing staff creates a demand for automation and the need for extracting more information from acquired data. Potential advantages of AI are already visible in screening routines in which a high number of patients (and associated data) are investigated for the presence or absence of disease, with results that are not worse than human performance. For example, McKinney et al. were able to show non-inferiority of their algorithm for screening of breast cancer as compared to experienced radiologists [6]. At the same time, AI results are being criticized because of the lack of transparency and consequently a potential lack of reproducibility [7].

The introduction of AI into the operation of radiology departments has led to optimizing resources [8]. Such operational AI should prove even more relevant in nuclear medicine (NM), which deals with radioactive isotopes, whose shelf-life is limited. Patient scheduling, management of preparation of radiopharmaceuticals, report generation and recovering and organizing previous NM and imaging studies are examples of tasks where AI could contribute to streamlining the operation of a department.

We must however admit that AI still has little place within NM so far. No doubt, this may be related to the fact that smaller patient numbers pass through a NM department every day as compared to, e.g. a radiology department. However, this is underestimating the potential of AI as on the one hand, each patient image represents numerous—be it correlated—data [9], and on the other hand, AI methodology has been shown to be able to adjust to smaller datasets by utilizing knowledge obtained in larger ones.

In this paper, we provide a short and concise review of the current state of the art in the field for both more physically and more clinically oriented components of AI applications in imaging. For more detailed reviews on each specific topic, readers are directed to other, more specialized articles [10–17]. Finally, some ideas on the introduction and use of AI in NM are discussed.

### Definitions

Throughout the literature, different terms and phrases are being used to describe the underlying principles of AI. In a 2018 communication by an independent high-level expert group set up by the European Commission, artificial intelligence refers to systems that display intelligent behaviour by analysing their environment and taking actions — with some degree of autonomy — to achieve specific goals [18]. We refer the reader to this document or other recent articles for comprehensive definitions of machine learning, deep learning and neural networks [1].

### The basics of AI: statistics

It is sobering to consider how AI is connected to classical, established statistical modelling. At the same time, it claims a right of its own since it mitigates a number of often overlooked but critical weaknesses in classical statistical procedures which are commonly used in the medical literature. Normal practice is to test the possible difference in the value of a biomarker (e.g. image intensity reflecting blood flow, oxygen extraction or metabolism) across two groups or conditions using a *t*-test. This statistical procedure quantifies the (un-)likelihood of the experimental data arising from identical distributions. However, it does not provide insight to the differentiability of the two groups based on the value of the biomarker. If a biomarker is claimed to be an early marker of disease, it is less relevant at which statistical significance level the values differ between prodromal patients and a control group than it is as to how accurately the marker itself differentiates between groups. Hence, a low *p*-value can lead to an artificially high confidence in a biomarker’s ability to actually detect disease. The statistical concept of *p*-values in itself must be interpreted with care because it tells little about how replicable a result is. In fact, it can be shown that if a statistical test yields a *p*-value of 0.05 (i.e. the standard level of significance), the probability of replicating this result is only 50% [19]. The probability increases only to 80% for a *p*-value ten times smaller, *p* = 0.005. Together with procedural flaws, such as serially adding variables to control for in regression, referred to as “researcher degrees of freedom,” it leads to an inflation of false-positive results and the ongoing replication crisis [20, 21].

In contrast, machine learning (ML) conceptually aims to optimize accuracy and reproducibility and in the case of AI even avoids human procedural bias in parameter selection. Interestingly, a ML approach does not necessarily venture beyond classical statistical modelling, it only takes a different approach to interpretation.

Linear regression is a classical statistical model but may also be seen as one of the simplest types of ML. Whereas in classical applications the interest is in determining the effect of certain covariates on a target, often reduced to a *p*-value as outlined above, the ambition of a ML application is to ensure the best possible fit to the observed data, and therefore predictive performance, while at the same time optimizing replicability.

AI diverts from classical statistics where it promotes more complex models in order to increase predictive accuracy. In particular, in case of deep learning (DL), classical statistical models such as logistic regressions are merged into hierarchies in such a way that the results of initial logistic regressions are used as input to others in so-called neural networks. This architecture implies that complex interactions between input variables are effectively utilized to increase model performance. It also implies that raw data, such as images, may naturally be input to the model, instead of summary features (such as for instance tumour volume, texture, etc.), which depend on human interaction.

Hence, AI, in comparison to classical statistical models, seeks to optimally identify feature differences between groups to optimize model performance while promoting generalizability beyond the study data initially available and has the ability to operate on raw healthcare data, such as images or health record information, without the need for manual feature extraction and summary, yielding a potentially more unbiased approach to knowledge discovery [22].

### Applications of AI in NM

For the sake of brevity and clarity, two major components in which AI can play a part can be discerned. The first component concerns image formation and image processing tasks and will be referred to as the “physics” component in the following sections. The second is largely application driven and hence will be referred to as the “clinical” component. It concerns routine workflow and final clinical endpoints, e.g. diagnosis, prognosis and prediction of response to therapy. Although these two components will be treated separately in this review, both are ultimately connected, the convolutional neural network (CNN) concepts being similar and very often associated within a single imaging paradigm. An example within this context may be the radiomics pipeline proposed by Hatt and Visvikis et al., where the development of AI-based image formation and segmentation algorithms serves to accurately determine regions of interest and extract imaging biomarkers which can subsequently be used in diagnosis and/or patient therapy stratification as well as follow-up [1, 23].

In addition, the available data from multimodality devices such as PET/CT or PET/MR and from the emerging total body PET technology is expected to largely increase with the development of (multi)parametric imaging. Within this context deep learning (DL)-based reconstruction and analysis, algorithms are potentially more efficient to deal with the increasing volumes of acquired data.

## Section 1: “Physics”

### Image reconstruction and data corrections

Following data acquisition, a conventional image reconstruction task involves the estimation of representation parameters for the accurate determination of the in vivo spatiotemporal radioactivity distribution. This involves the use of a model which accounts for different aspects including scanner/detector geometry, the physics of the detection process, physiological motion, measured data noise, etc. Until the late nineties, image reconstruction in NM was based on filtered-back projection. In current clinical practice, it is an iterative process that involves a measure of the difference between the modelled and the measured data. In the process, the difference is minimized through successive iterations using an objective function of choice (Poisson log likelihood). The introduction of AI to this reconstruction process represents a fundamental paradigm shift. The measured data are now mapped to an estimate of the image that we would like to see as a result. This is achieved by learning a reconstruction operator using training data. Data need to be sufficiently diverse and variable to cover all possible different imaging possibilities.

In the so-called direct reconstruction, the training is performed between the raw data in the form of sinograms/projections and the reconstructed images [24], with newer approaches using generative adversarial networks originally proposed in the context of image-to-image translation [25]. In the second scenario, DL may intervene within the conventional iterative image reconstruction process by denoising the successive image estimates or by introducing data based on prior information throughout the iteration process. Hence, it facilitates a faster convergence combined with a more accurate final image estimation [26, 27]. So far, the majority of these works have taken place in PET imaging and less in SPECT imaging [28]. Future work will need to provide further evidence of the advantages of using DL for image reconstruction tasks, in terms of the *(i)* reconstructed image’s qualitative and quantitative accuracy compared to state-of-the-art iterative algorithms, but also *(ii)* in terms of speed of execution which should be largely improved with “direct reconstruction” DL approaches (once the algorithm has been trained). This latter point can have a significant impact within the context of total body PET imaging where the volume of collected data increases substantially relative to current clinical PET imaging devices. Also, it will be necessary to specifically compare their performance to that of post-processing reconstruction image improvements based on DL such as denoising or super-resolution tasks.

In terms of data corrections, the majority of the work has been focusing on the field of DL-based attenuation correction (AC) and less on scatter correction (SC). The large majority of the proposed AC approaches are based on a wider image-to-image translation (also known as “image generation”) field of DL which is a generic task of AI [14]. The objectives of these developments are to produce CT equivalent–based AC maps from MR images in multimodality PET/MR [29], improve the attenuation maps produced by maximum likelihood reconstruction of activity and attenuation (MLAA) approaches [30] or totally remove the need for anatomical image information by directly producing attenuation-corrected images from non-attenuation-corrected PET data [31]. In terms of SC, the scatter sinograms could be produced from emission and attenuation raw data in PET or SPECT imaging [32, 33] or the direct production of scatter-corrected images (usually combined with AC) with non-corrected PET images being used as the input data to the network [34].

These developments show great promise, but there is a clear need for the evaluation of the robustness of these approaches both in terms of variability in anatomical location as well as presence of abnormalities, a study that can only be achieved by considering larger patient populations.

### Image processing and analysis

Again, one can identify two main areas: the one dealing with a post-processing improvement of the reconstructed images (remove noise, improve resolution) and the second with a further analysis of the data that allows the extraction of imaging biomarkers which subsequently can be used for different clinical endpoints [5]. Denoising primarily aims at pushing the limits of low-dose NM imaging and is based on the use of the low dose standard reconstructed PET images alone or in combination with anatomical modality images (predominantly MRI) as an input [26, 35]. DL-based super-resolution aims at obtaining a high-resolution image from a low-resolution input image. Approaches rely on training that uses either paired low/high-resolution NM images or combined low-resolution PET and high-resolution MR images [36]. Again, results are promising, but most studies lack a comparison with current state-of-the-art denoising techniques.

Concerning NM image analysis, the most significant developments deal with the field of image segmentation. NM images suffer from low spatial resolution and—as a result—partial volume effects which render the segmentation of functional volumes of interest a challenging task relative to anatomical image-based ROI definition. The first signs of the potential performance of DL-based segmentation algorithms relative to the current state-of-the-art have started emerging in the early segmentation software challenges organized in 2016 [37]. In this work, 150 acquired and simulated physical phantom datasets and 25 delineated 3D tumour volumes in PET images were used for training purposes. The CNN-based algorithm led to superior precision in the segmentation results, statistically outperforming 9 out of the 12 other segmentation approaches participating in the challenge, some of them among the current state-of-the-art. More recent results suggest that a 3D U-net type architecture based on encoder-decoder layers can provide high-accuracy functional tumour volume segmentation results from PET images in different cancer models and locations without the need for any user intervention [5]. In practice, however, results are not optimal yet. In a recent study in patients with cervical cancer by Pinochet et al., it became clear that the segmentations and total metabolic tumour volumes determined by their algorithm needed to be verified and, sometimes, even corrected to be similar to the manual segmentations [38]. Future direction of research in this field will concern the exploitation of multimodality datasets within the context of automatic image segmentation combining PET, CT and MR whenever available [39]. Almost all works in the field of image reconstruction and analysis referred to above have been carried out considering static acquisitions, but all of them can be equally applied to dynamic imaging, including direct parametric PET image reconstruction [40].

Finally, another significant future application for NM imaging, within the wider area of DL-based image synthesis (mentioned in the data correction section above), is that of image harmonization. Multicentre trials will become even more important in the future given the requirements for large training datasets in AI developments. Within this context, it is essential to ensure the development of harmonization approaches which go beyond acquisition and processing standardization protocols that minimize but do not eliminate imaging technology-related variability or otherwise known as the “centre effect” [41]. Early evidence suggests that DL, and more specifically the field of image-to-image translation using generative adversarial networks (GANs), can play a major role in image-based harmonization methodologies [42].

A field where AI may play an important role also is image registration. Despite the fact that in clinical practice there is already a strong clinical multimodality component (PET/CT, SPECT/CT, PET/MR) in use, the application of AI in image registration can be quite relevant. Consider longitudinal studies but also the alignment of PET and CT data within a single PET-CT study. Patients move, and there is always some mismatch between CT and PET, either random, systematic or cyclic. Also, AI may help in metal artifact reduction strategies for improved attenuation correction in hybrid PET/CT imaging.

## Section 2 — “Clinical”

### Neurology

Neurodegenerative disease is a major application of NM. It is common experience that reading these brain images (FDG-PET, DaT-scan, amyloid PET) can be quite challenging. Consequently, aid from AI has been sought. In a proof-of-concept study, Kim et al. described the use of a network in discriminating between Parkinson’s disease and a normal distribution of ^123^I-ioflupane. Although the study was relatively small (118 abnormal and 63 normal scans) and of a retrospective character, they were able to show a high test accuracy with a ROC of 0.87 [43]. Choi et al. built a DL model for discriminating the FDG uptake patterns in Alzheimer’s from normal controls. That model was then transferred to images of patients with mild cognitive impairment to see if it could discriminate those patients that would rapidly convert to Alzheimer’s disease from others. Also, they studied whether patients with Parkinson’s dementia could be discerned. They concluded that their DL-model could successfully be transferred to multiple disease domains [44]. Similarly, Son et al. trained a DL system in interpreting ^18^F-florbetaben images and concluded that in visually equivocal scans, their algorithm was able to discriminate between progressive disease and negative cases, thus providing information on clinical outcome and even prognosis assessment [45]. Finally, the use of deep learning algorithms may possibly expand FDG-PET/CT to other diseases, e.g. to discriminate between ALS and ALS mimics [46].

Such examples from the literature show the potential of AI in relatively small patient population studies. Thorough validation and finetuning on larger patient populations are needed to facilitate clinical translation.

### Cardiology

Myocardial perfusion imaging (MPI) using SPECT or PET is the most often performed scan in nuclear cardiology. The aim of this examination is to help the clinician to determine whether the patient suffers from cardiac disease (usually coronary artery disease), whether treatment is necessary and, if so, which therapy is preferred (revascularization vs. optimal medical treatment). The most commonly used modality is SPECT using Tc-99 m-labeled perfusion tracers. Due to the high number of examinations and the relatively standardized procedure of the test, myocardial perfusion imaging using SPECT is an interesting model for applying AI. The examination is usually carried out on a hybrid device, so that a CT dataset (or in the case of PET sometimes an MRI data set) is also generated.

The first applications of AI in nuclear cardiology were the segmentation of imaging data, the diagnosis of obstructive coronary artery disease and the prediction of major adverse cardiac events (MACE). It has already been shown that the automatic determination of the valve plane — an important step in the reconstruction of image data, often requiring user interaction with the respective software — was performed by an AI approach in a manner comparable to highly experienced NM specialists [47]. Also, a work on datasets of almost 1000 patients showed that a fully automated analysis of the MPI studies showed a good agreement with experts [48]. In another study, besides the categorization of patients based on MPI data regarding the presence of obstructive CAD, parameters were automatically selected from 33 clinical and quantitative data with an information gain [49]. Using this information, a prediction model was then trained with the help of the tenfold cross-validation technique, and the results were compared with the assessment of two experts, whereby the machine learning algorithm was not inferior. Similar results could be reproduced in other studies [50, 51]. The last major field of application is the attempt to prognosticate the further course of the disease. In contrast to the normal (human) approach where mainly the imaging data are used, AI allows the consideration of a large number of variables. In a work on more than 2500 patients, an ML algorithm for the prediction of MACE was trained and evaluated [52]. A total of 70 clinical, ECG or imaging parameters were provided to the algorithm. The prediction of MACE was significantly better when these parameters were used in combination rather than when only the imaging parameters were provided by the algorithm. Interestingly, the machine learning approach was even better than the interpreting physicians, who had access to all patient data [52].

These are promising examples to the use of AI in nuclear cardiology. Further developments are expected, e.g. in the reconstruction of imaging data. And it is also expected that there will be AI-based software tools available in the near future to support physicians in the interpretation of imaging data in everyday clinical routine. A position paper on the applications of AI for NM multimodality cardiovascular imaging has been recently published by EANM and EACVI [53].

### Oncology

In oncology, the movement towards personalized medicine is most clearly visible. It should tailor the treatment towards the genetic composition of the tumour(s) and take into account the extent of the disease and the condition of the patient. It is generally believed that AI will help in this process.

This is not limited to positron emission tomography alone. One of the least expected areas to introduce AI is in classic bone scanning with [^99m^Tc]Tc-labelled diphosphonates. However, bone scanning is much used, and interpretation can be difficult at times. Zhao et al. developed a deep neural network algorithm based on over 9500 planar bone scans, validated it on over 1250 cases and tested it also in over 1250 cases. Then, a competition was held between AI and 3 experienced NM specialists. In the initial part, AI reached to an overall diagnostic accuracy of 93%. In the second part, the algorithm performed slightly better than the physicians, proving non-inferiority. Time gain was enormous: the AI took just over 11 s to analyse, whereas the 3 physicians took on average 2 h and 15 min for the task [54]. Different algorithms for this task are being tested currently, and the early results show that despite the differences in approach, outcomes are quite comparable [55]. However, one also has to realize that a thorough validation in case of bone scanning is very difficult as a gold standard is lacking. Also, the algorithms used should be open to the public, lest they can be checked and challenged by others.

Another promising field of the application of AI is in response evaluation with ^18^F-FDG-PET/CT through extracting quantitative information (“radiomics”) from the images, which will be discussed in more detail below [56]. Very interesting is the hypothesis that the extraction of radiomics features may endorse a histological diagnosis or even replace a histological diagnosis when it is difficult or often impossible to get a biopsy [57]. A suggestion that goes beyond FDG, but to date, a very limited number of radiomics studies have considered other tracers.

### Diagnosis, radiomics, response to therapy and dosimetry

Traditionally, the extraction of “handcrafted radiomics” requires manual segmentation of the region of interest. Subsequently, thousands of human-defined and curated quantitative features from the ROI which for example describe tumour shape, intensity and texture are extracted [57–59]. As already mentioned in the “physics” section above, all of these steps can be performed by using AI. In terms of classification and model building, machine learning techniques have been used over the past 30 + years with variable levels of success and have been recently evaluated within the context of radiomics [60]. The objective is to identify those imaging features that are associated or causally related with a given clinical or biological endpoint. Latest evidence suggests that a consensus based on the results provided by multiple such techniques could improve a model’s prediction performance and reduce the variability of the results observed [61–63]. One current trend with DL involves the “direct model” building based on the use of identified/segmented volumes without having to explicitly extract radiomics features, a process known as the use of “deep features” [64, 65]. Preliminary works exist extracting these deep features from whole body images within the region of interest and associated segmentation steps embedded in the overall process. If successful, such an approach could allow the elimination of the time-consuming lesion identification and annotation/segmentation step, facilitating the introduction of image-derived features in the clinical workflow for diagnostic and therapy stratification purposes. One major challenge in such data-intensive AI applications is the need to exploit multicentre data, an essential step in bringing into routine clinical workflow single centre predictive and prognostic models in the future. The use of multicentre trials is associated with increased variability due to different image reconstruction protocols implemented on different manufacturer equipment as well as non-standardised data acquisition protocols. The approaches to handle such variability are either (i) based on allowing an AI model to learn from it or (ii) develop image harmonisation approaches based on AI, a field currently very much in the forefront of methodological developments [66].

Another area of application where AI could play a significant role is related to radionuclide dosimetry. Especially in children and young adults undergoing repeated scans, dosimetry and dose reduction are an issue. In a recent study by Wang et al., they showed that the introduction of AI has a potential to reduce the FDG-dosage up to a 1/8 dose equivalent (0.18 MBq/kg) yet providing interpretable images. SUV was not affected [67].

Based on a series of planar or 3D images, different dosimetry methods have been developed to calculate the absorbed dose distribution, which in turn can be used to quantify the whole-body dosimetry of the therapeutic agent and to support treatment planning [68]. Despite the potential of quantifying a companion diagnostic tracer for underpinning radioligand therapy (RLT) treatment planning, the estimation and extrapolation of the pharmacokinetics are associated with certain practical difficulties. Furthermore, the estimation of pretherapy pharmacokinetics normally requires multiple whole body dynamic scanning repeatedly for as long as possible. Although these issues may be resolved with the development of total body PET systems, such protocols are currently incompatible with routine clinical practice. Because of these technical limitations, pre-therapy imaging is usually only used to qualitatively select candidates for RLT and to rule out obvious risks. The patients are still treated with a fixed radiopharmaceutical activity [69]. AI may unveil the complex relationship between pre-therapy patient data, such as imaging, demographic data and laboratory data and the radiation dose distribution to be obtained during therapy. To illustrate this, a DL method was developed to automatically detect and segment prostate cancer lesions on ^68^ Ga-PSMA PET/CT-images [70], which may support the definition of the treatment targets for the planning. Furthermore, the potential of AI in predicting organ-wise or voxel-wise post-therapy dosimetry of radiopharmaceuticals has also been shown [71]. GANs have been developed to discover the complex relationship between pre-therapy patient data and post-therapy radiation dose distribution and therefore may predict the voxel-wise dose distribution for treatment planning. Finally, training of an AI dosimetry prediction model requires accurate dose estimation. DL methods have been developed to replace Monte-Carlo simulation for the estimation of voxel-wise dosimetry [71, 72]. Initial results indicate that these methods are computationally efficient to consider individual tissue density distributions as well as heterogeneity of the concentration of the radiopharmaceutical to improve dosimetry estimation from SPECT or PET measurements.

In summary, using AI in NM multimodality imaging has the potential to play a significant role in routine diagnosis but also in the more challenging personalised patient therapy monitoring and response assessment applications. Within this context, AI may play a role in the development of new radiotracers for imaging and therapeutic purposes [73]. In addition, AI can facilitate accurate voxel-wise dose calculations for dosimetry purposes in radionuclide therapy. Although the development of large axial field of view PET scanners may bring dynamic and parametric whole-body imaging to the forefront, the application of AI in this field is currently in its infancy, and the associated clinical interest remains to be demonstrated.

## Challenges for transferability from research to clinical practice

Despite the promises of AI, some of them outlined above, there are also clear limitations in terms of their implementation and most importantly in terms of their clinical acceptability. A recent review by Torres-Velazquez et al. discussed in detail all of these challenges for the use of DL in medical imaging [5].

First, there is the level of performance, as well as its reproducibility and robustness to variable imaging protocols and devices, which depends on the quantity and on the quality of the available data for training of the system. In addition, once the training of an algorithm has been completed, datasets that were not used in the training are needed to ensure validation and testing processes. Irrespective of the step (training, validation, testing) in the process, all data need to be accurately annotated since AI learns from this annotation and therefore depends largely on the precision and consistency of the available data. This cannot be emphasized enough and is a plea for structured reporting. Another solution here will be the use of consensus annotation from multiple observers/operators in order to reduce the potential impact of a single reduced quality annotation. This is critical for applications such as lesion detection tasks. Using actual clinical progression or outcome is a solution for other problems, such as predicting disease progression and response to therapy. In addition, quality control protocols for the evaluation of such annotations should be put in place. Finally, in combination with standardization initiatives, image-based harmonization approaches as mentioned above hold the potential to eliminate the impact of a ‘centre-effect’ by bringing data from multicentre trials together in an efficient manner for AI training purposes.

The development of AI normally depends on the availability of a large amount of data. Compared to other disciplines, it is usually challenging to gather such large cohorts of patients in NM. Apart from the variations of scanners or imaging protocols, NM imaging and therapy have additional variations in radiopharmaceuticals and dosages. This large complexity makes NM studies susceptible to incomplete or missing data. As a result, it hampers extensive development of AI methods in NM. There are some potential ways out. Physiologically based pharmacokinetic modelling may assist the translation of different radiopharmaceuticals during AI development [74]. And weakly supervised deep learning may accelerate the learning on incomplete datasets [75].

Another issue is the variability with respect to the targeted task. Lack of variability will lead to overfitting of the training data and poor generalizability. The developed approaches will work during training but demonstrate reduced performance during the validation/testing steps. Therefore, cross validation is a *conditio sine qua non* when developing models. But beyond this extensive clinical testing is necessary. In general terms, a minimum of 70% training and 30% validation is used. In the cross-validation approach, the k-fold cross-validation is the one most frequently used [5]. Data augmentation can be used in certain cases by e.g. shifting, zooming or rotating images [76]. Be aware that — in tasks with a clinical endpoint — such data augmentation approaches may be less efficient than when used for more physics-oriented tasks. Another approach is ‘transfer learning’, where the context of the specific issue of data quantity involves a first-level training of the AI on other types of available data not specific to the task in hand, with a fine-tuning step using the task-specific datasets. The same concept can be used also for domain adaptation where a successful network within a given task can be used in another domain (e.g. moving from PET to SPECT image segmentation).

For the testing and validation of AI algorithms, including adherence to international and national standards, large datasets are needed. This inevitably means (standardized) multicentre cooperation and sharing of data, certainly within the field of NM and molecular imaging. Given the difficulty in data sharing, e.g. due to the Directive 95/46/EC (General Data Protection Regulation) of the EU, different approaches can be highlighted and encouraged. One concerns the creation of large, publicly available, open access image databases, such as for example ADNI or TCIA [77, 78], which preferably should be encouraged by public organisations. In this respect, the upcoming European Health Data Space is also eagerly awaited. Other variability aspects that need to be addressed within such publicly available repositories are the availability of external parties to the imaging data (such as demographics, omics, histopathology, outcome). Alternatively, the potential training of a given model could go through distributed training [79], where a model is further refined by exploiting local databases without the need for the exchange of datasets in a larger repository, hence reducing reglementary-related obstacles. In both cases, strict cybersecurity protocols need to be adhered to, not only in terms of data but also in terms of algorithm security in order to ensure their widespread deployment, usage and reliability.

## Explainable AI

A key dimension to ensure the use of AI is user trust from both the medical professional and the patient. They should be able to understand the AI’s answers (both in real-time and afterwards) and should be able to determine when to trust the AI and when the AI should be distrusted. This will be an essential element for clinical transferability. Current approaches to AI have a high ‘black box’ factor, which is a clear drawback for clinical adoption. The different approaches need explainable/interpretable models based on DL in order for clinicians to understand, interpret and therefore trust such models. Different approaches have been proposed, and their large-scale evaluation is clearly a future challenge. Most of them involve the use of a retro-propagation of information from the results to the input data with different visualisation possibilities [79, 80].

With this in mind, the concept of Explainable AI (XAI) was introduced. XAI emphasizes the need that the response should be understandable: why did the algorithm arrive at a certain conclusion? Does it make sense? The right for explanation is seen as a basic requirement in our society and is also a request for certification (CE marked and FDA approved). Blind reliance on AI may lead to Kafka-like situations, as depicted in the New York Times bestseller ‘Weapons of Math Destruction’ by Cathy O’Neal [81]. Why should a patient accept receiving adjuvant chemotherapy if both the patient and the doctor do not understand the reasons for doing so?

There are 2 routes to approach this problem, viz*.* the route of the interpretable model or the route of the explainable model. An interpretable model provides its own explanation and can be intuitively understood, e.g. the risk of treatment-induced toxicity is higher in elderly patients. In contrast, an explainable model does not provide its own explanation. These models are black-box models and are typically too complicated to be understood by humans. Additional techniques or visualization tools (e.g. an activation map on an image) are needed to understand how the model made the predictions. This approach is also seen as a quality assessment. For example, if an AI model concludes that a lung nodule is malignant but the most informative image voxels are outside the nodule, then the likelihood of a false positive finding is higher.

Another consideration is *infobesity* (overload of information) whilst typically the human brain can handle a maximum of 5 variables to make a decision. The challenge is to have ‘glass box’ models that are explainable to a ‘human-in-the-loop’, without sacrificing AI performance. There is also the request from users to have a bidirectional flow of information, rather than the AI giving a conclusion unidirectionally. A frequent requirement is a user interface allowing to ask questions on the answers/predictions of the AI model, to put them into context, to compare to previous cases, etc. Methods that can provide qualitative (visual) feedback through the use of network visualization techniques using saliency/activation maps can be handy therefore to accompany these developments [64, 80]. This user interface could also be used several years later in e.g. retrospective evaluation of the AI performance, or in case of medico-legal issues.

Again, the European Union is taking up the gauntlet by proposing a regulatory framework for AI (https://digital-strategy.ec.europa.eu/en/policies/regulatory-framework-ai) with (a.o.) the goal that Europeans can trust what AI has to offer.

## Comparing performance of algorithms

As already mentioned earlier, over the past 3–4 years, there have been numerous DL algorithms and associated pipelines proposed for different tasks in NM imaging. One of the difficulties with DL algorithms are the hundreds of parameters that need to be optimized but also the different types of networks and their implementation details in terms of e.g. the number of layers and associated connections, optimizers and loss functions that can be varied. Consequently, it is crucial to compare the performance of all these various implementations under controlled conditions. Given all the potential variations, it is impossible to reproduce results from the literature by re-using the proposed approaches. Alternative verification methods are to be implemented, such as the creation of software challenges. In these challenges, a given dataset is made available for developers to evaluate the performance of their algorithms within a controlled environment. Numerous software challenges have been organized throughout the years, mainly within the field of image segmentation [37], but also in other fields of interest increasingly targeting clinical endpoints (https://grand-challenge.org/challenges/). Whereas NM has been lagging behind, it is the conviction of us that such challenges can and should be organised in a multicentre set-up. Challenges should be based on a predetermined categorisation of potential applications using different factors, such as impact and potential utility and uptake in clinical practice. Different points may be investigated by such challenges, such as for example:

1. Explain the need for data and associated requirements (volume, annotation, QC), definitions of training and validation datasets
2. Mention the dependency of data volumes needed on the targeted task, which also requires standardisation and harmonisation for the exploitation of multicentre datasets
3. Define algorithm-related aspects like the number of parameters, optimisation protocols, robustness, transferability on datasets from different instruments or body locations
4. Deal with model interpretability (white–grey box concept compared to black box), and thus with the acceptability by professionals (medical physicists, physicians) and the public (patients and families)
5. Integrate established domain knowledge (e.g. PBPK modelling) in AI algorithms or training procedure to reduce the requirement of data volume and improve the robustness of the algorithms
6. Deal with training issues for implementation in clinical practice (computational burden, periodical updating)

When developing challenges and algorithms, it is advisable to bear the best practices for algorithm development as recently published by Bradshaw et al. [82].

## Conclusions

The time to implement AI in medicine is now. Nuclear medicine and molecular imaging are no exception to that development. AI shows great promise to improve image quality, to personalise dosages (both in diagnosis and theranostics) and to help in image interpretation. It opens ways to fully exploit the potential of NM (which by nature is a numerical specialty), an aspect that has gathered momentum over the past decade with the advent of radiomics. As such, AI has the potential to improve clinical workflows that will increase overall efficiency but also facilitate personalised medicine for the benefit of a patient. Here, we should not forget the introduction of total body scanners which cause an enormous increase of data to be handled. It seems obvious that AI and total body scanners are natural partners to tackle this problem.

In this paper, we have highlighted the current state of the art in AI for NM. Research in AI for NM has made great strides over the past couple of years as this paper has described. We have also highlighted areas where work needs to be concentrated in order to improve overall acceptability of AI by the NM community.

In order to move forward with clinical adoption, the validation of outcomes/results of individual trials/studies is needed, and this is where multicentre and multigroup cooperation is of the utmost importance. Comparing the performance of algorithms through challenges is needed considering both methodological and clinical outcomes. Demonstrating the potential interest of AI in NM through these challenges represents also an essential element for its adoption within our field. As with any new technology (and even more true for software related) developments, the field requires also industrial partners to be proactive in facilitating its clinical implementation.

Last but not least is education. The transmission of knowledge for the implementation of AI in NM and molecular imaging is dearly needed. These educational programs should target both the scientific and clinical aspects. Such educational programs will ensure that current and next-generation scientists and clinicians will become familiar and grow-up with AI and therefore enhance further its potential adoption and development.
